# Constrained body shape among highly genetically divergent allopatric lineages of the supralittoral isopod *Ligia occidentalis* (Oniscidea)

**DOI:** 10.1002/ece3.1984

**Published:** 2016-02-09

**Authors:** Carlos A. Santamaria, Mariana Mateos, Thomas J. DeWitt, Luis A. Hurtado

**Affiliations:** ^1^ Department of Wildlife and Fisheries SciencesTexas A&M UniversityCollege StationTexas; ^2^ Biology FacultyCollege of Arts and SciencesUniversity of South Florida Sarasota‐ManateeSarasotaFlorida

**Keywords:** Allometry, body shape, coastal biodiversity, cryptic species, leave‐one‐out cross‐validation, Oniscidea, test of phylogenetic signal

## Abstract

Multiple highly divergent lineages have been identified within *Ligia occidentalis sensu lato*, a rocky supralittoral isopod distributed along a ~3000 km latitudinal gradient that encompasses several proposed marine biogeographic provinces and ecoregions in the eastern Pacific. Highly divergent lineages have nonoverlapping geographic distributions, with distributional limits that generally correspond with sharp environmental changes. Crossbreeding experiments suggest postmating reproductive barriers exist among some of them, and surveys of mitochondrial and nuclear gene markers do not show evidence of hybridization. Populations are highly isolated, some of which appear to be very small; thus, the effects of drift are expected to reduce the efficiency of selection. Large genetic divergences among lineages, marked environmental differences in their ranges, reproductive isolation, and/or high isolation of populations may have resulted in morphological differences in *L. occidentalis*, not detected yet by traditional taxonomy. We used landmark‐based geometric morphometric analyses to test for differences in body shape among highly divergent lineages of *L. occidentalis*, and among populations within these lineages. We analyzed a total of 492 individuals from 53 coastal localities from the southern California Bight to Central Mexico, including the Gulf of California. We conducted discriminant function analyses (DFAs) on body shape morphometrics to assess morphological variation among genetically differentiated lineages and their populations. We also tested for associations between phylogeny and morphological variation, and whether genetic divergence is correlated to multivariate morphological divergence. We detected significant differences in body shape among highly divergent lineages, and among populations within these lineages. Nonetheless, neither lineages nor populations can be discriminated on the basis of body shape, because correct classification rates of cross‐validated DFAs were low. Genetic distance and phylogeny had weak to no effect on body shape variation. The supralittoral environment appears to exert strong stabilizing selection and/or strong functional constraints on body shape in *L. occidentalis*, thereby leading to morphological stasis in this isopod.

## Introduction

Morphological stasis, the lack of change in gross external anatomy over long periods, is conspicuous in the fossil record and among extant taxa (Gould and Eldredge 1977; Wake et al. 1984; Bickford et al. 2007). Considered one of the most challenging problems in evolutionary biology, this phenomenon is central to understanding the gradualism versus saltation discrepancy (Gould and Eldredge 1977; Charlesworth et al. 1982; Wake et al. 1984; Futuyma 2009). Cryptic diversity, which is pervasive in nature (Bickford et al. 2007; Trontelj and Fiser 2009), provides remarkable opportunities to study the extent and underlying causes of morphological stasis. Studies of extant cryptic species have revealed morphological stasis among lineages that appear to have diverged long ago (e.g., up to tens of millions of years) and occupy distant geographic areas (Lee and Frost 2002; Lavoue et al. 2011). Nonetheless, in certain taxa formerly regarded as examples of morphological stasis, the application of geometric morphometric approaches uncovered previously unknown and lineage‐diagnostic divergence in external morphology (Strumbauer and Meyer 1992; Maderbacher et al. 2008; van Steenberge et al. 2015). Therefore, variation in gross external morphology must be carefully evaluated before a taxon is deemed “morphologically static.”

Members of the semiterrestrial isopod genus *Ligia* appear to have experienced morphological stasis. DNA sequence data have revealed high levels of isolation and genetic allopatric differentiation within recognized coastal species of this genus from separate regions (i.e., Hawaiian archipelago, Gulf of California‐adjacent areas, Caribbean, and Mediterranean), each of which is suggested to harbor a cryptic species complex (Hurtado et al. 2010; Santamaria et al. 2013, 2014; Hurtado et al. unpublished). Traits such as direct development (Carefoot and Taylor 1995), which restrict coastal species of *Ligia* to a vertically narrow strip of the coast (i.e., rocky upper intertidal and supralittoral), have likely contributed to these high levels of allopatric genetic differentiation (Hurtado et al. 2010). Members of *Ligia* are among the few animals that have adapted to live exclusively within this environment, which is characterized by extreme conditions (Hurtado et al. 2013). These include regular exposure to a broad range of temperatures, air humidity, and water salinity levels (from rain, wave splash, storm surge, and tides), and to predation by terrestrial, aerial, and marine animals (Menge 1976; Brown 2001; Ellis et al. 2007; Castilla et al. 2008; Donahue et al. 2009). Such conditions might impose strong stabilizing selection on morphology, thereby limiting the morphological divergence that can accompany genetic differentiation.

**Figure 1:**
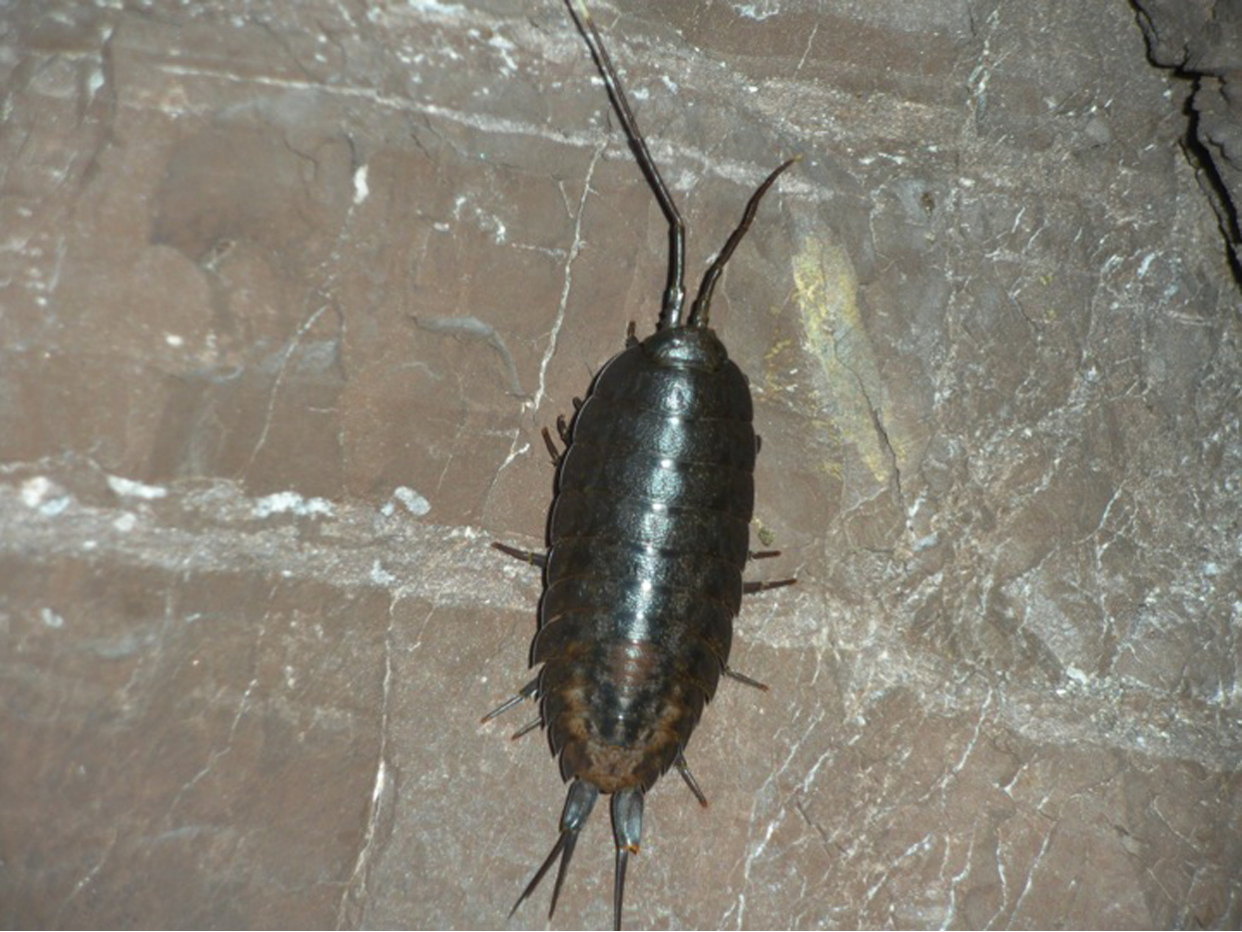
Individual of *Ligia occidentalis* on a rock (Photo by L. A. Hurtado).

The rocky intertidal isopod *Ligia occidentalis* (Dana 1853) represents the most striking example of morphologically cryptic diversity reported to date within the genus (Fig. 1). It is distributed on the Pacific coast of North America from southern Oregon to Central Mexico, including the Gulf of California. This isopod is currently recognized as a single species for which no junior synonyms have been proposed (Espinosa‐Pérez and Hendrickx 2001; Schmalfuss 2003). Phylogeographic analyses of *L. occidentalis*, however, revealed the existence of highly divergent lineages that likely represent a cryptic species complex (hereafter *L. occidentalis* sensu lato). Divergences among lineages are as high as 29.9% (Kimura‐2‐parameter; K2P) for the Cytochrome Oxidase I gene (COI), where > 60% of pairwise comparisons among localities exhibit COI K2P divergences > 10% (Hurtado et al. 2010). Deep divergences among lineages suggest a long evolutionary history of *L. occidentalis s. l*. in the region, possibly since the Miocene, whereas high levels of genetic differentiation among populations indicate gene flow is severely restricted, even over small geographical distances, implying long‐standing isolation of populations (Hurtado et al. 2010). Crossbreeding experiments suggest postmating reproductive barriers may exist among some lineages (McGill 1978), and surveys of mitochondrial and nuclear gene markers do not show evidence of hybridization among divergent lineages (Eberl et al. 2013; Hurtado et al., unpublished data).

Divergent lineages of *L. occidentalis s. l*. are exposed to markedly different environmental conditions, which could entail natural selection promoting morphological divergence. *Ligia occidentalis s. l*. is distributed along a ~3000 km latitudinal gradient that encompasses several proposed marine biogeographic provinces and ecoregions in the eastern Pacific (Spalding et al. 2007; Robertson and Cramer 2009; Briggs and Bowen 2012). In general, the eight main lineages have nonoverlapping geographic distributions, with distributional limits that generally correspond with sharp environmental changes. The geographical limit between the two most divergent Pacific clades of *L. occidentalis s. l*. (20–25% divergence for COI) distributed from southern Oregon to the southern Baja California Peninsula occurs at the Point Conception biogeographical boundary, which separates the Oregonian and Californian marine zoogeographical provinces (Eberl et al. 2013). The geographical limit between these two main clades largely reflects the changes in sea surface temperature that define the Point Conception biogeographical boundary along the shores of both the mainland and the Northern California Channel Islands (Eberl et al. 2013).

Within the Gulf of California, sharp environmental differences are also observed in the distribution of the two most divergent lineages of *Ligia* (15–26% K2P COI) occupying this basin (Hurtado et al. 2010). One lineage occurs in the Northern Gulf of California (from the mouth of the Colorado River to the Midriff islands), whereas the other is distributed in the Southern Gulf of California. The Northern Gulf is characterized by strong seasonal variation in water temperatures (>30°C in the summer; 8–12°C in the winter), large tidal regimes (up to 10 m), and high summertime salinities (35–40 ppt), whereas the Southern Gulf is characterized by somewhat lower salinities, smaller tidal regimes, and moderate seasonal variation in water temperatures (30–32°C in the summer; 18–20°C in the winter) (Brusca 2007 and references therein).

Large genetic divergences among lineages, reproductive isolation among them, high isolation of populations, and/or marked environmental differences in their ranges may have promoted morphological differentiation in *L. occidentalis s. l*. not detected yet by traditional taxonomy. Use of more sophisticated approaches, such as geometric morphometrics, may detect differences among lineages of this isopod. Geometric morphometric analyses have been useful for discriminating cryptic lineages of other crustaceans (Silva et al. 2010a,b; Zuykova et al. 2012), as well as in an array of other animal taxa (e.g., Carvajal‐Rodríguez et al. 2006; Francuski et al. 2009; Milankov et al. 2009; Mitrovski‐Bogdanovic et al. 2013; Schmieder et al. 2015). Remarkable body shape differences can be attained rapidly in response to environmental variation (e.g., the freshwater isopod *Asellus aquaticus*; Eroukhmanoff and Svensson 2009). Body shape is one of the most important and comprehensive features of an organism's phenotype (Ingram 2015), and is relevant to ecological traits of isopods (e.g., Schmalfuss 1984; Broly et al. 2015). The presence of a rigid exoskeleton in crustaceans facilitates unambiguous placement of landmarks for geometric morphometrics analyses of body shape.

In this study, we used landmark‐based geometric morphometric analyses to test for differences in body shape among highly divergent lineages of *L. occidentalis s. l*., and among populations within these lineages. We used landmarks that captured taxonomically informative regions that have been used to distinguish *Ligia* species. We conducted discriminant function analyses to test whether body shape morphometrics can be used to diagnose genetically differentiated lineages of *L. occidentalis s. l*. We used thin‐plate‐spline transformations to describe the general shapes of individuals within lineages and make comparisons among lineages. We also tested (1) for associations between phylogeny and morphological variation, and (2) whether genetic divergence is correlated to multivariate morphological divergence. Our study contributes to understanding the constraints on morphological evolution in a cosmopolitan genus characterized by high levels of cryptic diversity and an extreme habitat (i.e., the supralittoral).

## Materials and Methods

### Samples

**Figure 2:**
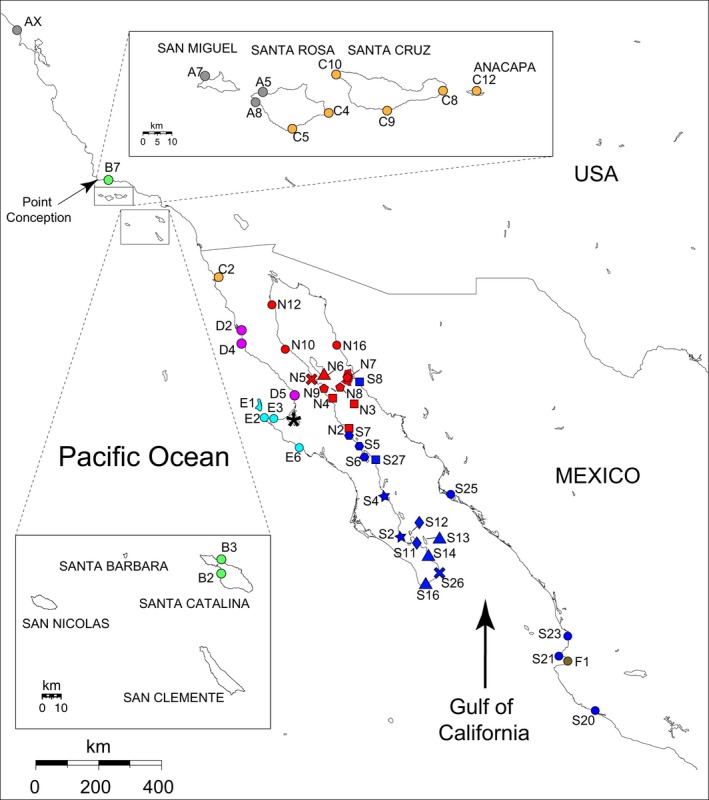
Map of Sampled localities. Color and shape correspond to those in Hurtado et al. (2010). AX‐”Berkeley”; A5‐N.W. Talcott; A7‐Otter Harbor; A8‐Fossil Reef; B2‐Little Harbor; B3‐Ithsmus Cove; B7‐Refugio; C2‐Corona Ensenada; C4‐East Point; C5‐Johnsons Lee; C8‐Smugglers Cove; C9‐Willows; C10‐Fraser Cove; C12‐Frenchy's; D2‐San Quintin; D4‐Arroyo Ancho; D5‐Tomatal; E1‐Cedros Is.; E2‐Punta Eugenia; E3‐ El Queen; E6‐San Hipolito; F1‐Vallarta; S2‐Cajete; S4‐San Cosme; S5‐Mulege; S6‐ Bahía Armenta; S7‐San Lucas; S8‐Bahía Kino; S11‐ La Paz; S12‐Isla Espiritu Santo; S13‐Isla Cerralvo; S14‐Barriles; S16‐Cabo San Lucas; S20‐ Boquita; S21‐Punta Mita; S23‐Aticama; S25‐Topolobampo; S26‐Cabo Pulmo; S27‐San Nicolas; N2‐Santa Rosalia; N3‐Isla San Pedro Martir; N4‐San Francisquito; N5‐Bahía de los Angeles; N6‐Isla Angel de la Guarda (2 localities); N7‐Isla Tiburon (2 localities); N8‐Isla San Esteban; N9‐San Rafael; N10‐San Luis Gonzaga (2 localities); N12‐San Felipe; N16‐Puerto Libertad. * denotes Guerrero Negro Lagoon. Modified from Hurtado et al. (2010).

We analyzed a total of 492 *Ligia* individuals from 53 Pacific localities distributed between central California and central Mexico, including the Gulf of California (Fig. 2; Table 1). Samples were collected by hand during 2007–2010 and stored in 100% ethanol under permits from California Department of Fish and Game (USA) No. 9881, and Comisión Nacional de Acuacultura y Pesca (Mexico) No. DGOPA.l0337.020908.2952. Once in the laboratory, they were stored in ethanol at −80°C until dissection. These specimens were part of the original collections obtained for the *Ligia* phylogeographic study of this region by Hurtado et al. (2010) and represent the eight main highly divergent lineages (clades) identified in that study, which, for the most part, have nonoverlapping distributions (Hurtado et al. 2010; Eberl et al. 2013). For consistency among studies, we use the same names for these lineages as in Hurtado et al. (2010). (1) A Central California clade (*Clade A*; gray in Fig. 2) distributed from southern Oregon to north of Point Conception, CA, and some localities of the Northern Channel Islands, mainly in the western part (Eberl et al. 2013). (2) A Southern California clade (*Clade B*; light green in Fig. 2) found on the mainland from south of Point Conception to San Diego, and in Santa Catalina Island. (3) A California clade (*Clade C*; orange in Fig. 2) distributed from San Diego, CA to Ensenada, Mexico, and in some localities in the eastern part of the Northern Channel Islands. (4) A Baja Pacific North clade (*Clade D*; magenta in Fig. 2) distributed from Ensenada, Mexico, to north of the Guerrero Negro Lagoon. (5) A Baja Pacific South clade (*Clade E*; light blue in Fig. 2) found from south of this lagoon to Puerto San Carlos, Mexico. (6) A Careyes Clade (*Clade F*; brown in Fig. 2) reported only from populations in Puerto Vallarta and Careyes, Mexico. (7) A Gulf North clade (*Clade N*; red in Fig. 2), which includes populations in the northern Gulf of California, including localities in the midriff islands. (8) A Gulf South clade (*Clade S*, blue in Fig. 2) distributed in the southern Gulf, and mainland south of the Gulf to the State of Guerrero.

**Table 1:** Sampled localities by lineage with corresponding sample size. Label IDs correspond with those in Fig. 2

	Population	*N*	Label ID
Clade A *N* = 38	Berkeley	9	AX
	Fossil Reef	10	A8
	N.W. Talcott	9	A5
	Otter Harbor	10	A7
Clade B *N* = 31	Ithsmus Cove	9	B3
	Little Harbor	10	B2
	Refugio Beach	12	B7
Clade C *N* = 61	East Pt. Beach	10	C4
	Ensenada (Corona Beach)	10	C2
	Fraser Cove	6	C10
	Frenchy's	9	C12
	Johnson's Lee Beach	8	C5
	Smugglers Cove	10	C8
	Willows’ Anchorage	8	C9
Clade D *N* = 33	Arroyo Ancho	10	D4
	South of Quintin	14	D2
	El Tomatal	9	D5
Clade E *N* = 37	I. Cedros	9	E1
	Punta Eugenia	8	E2
	Campo Queen	10	E3
	San Hipolito	10	E6
Clade F *N* = 9	Puerto Vallarta	9	F1
		
Clade N *N* = 137	Angel de la Guarda	10	N6
	Bahia de los Angeles	10	N5
	Puerto Libertad	10	N16
	Ratolandia	10	N7
	San Esteban	17	N8
	San Felipe	10	N12
	San Francisquito	9	N4
	San Rafael	10	N9
	San Luis Gonzaga	10	N10
	San Luis Gonzaga (Mudflat)	4	N10
	San Pedro Martir	12	N3
	Santa Rosalia	9	N2
	El Tordillo	9	N7
	Viborita	7	N6
Clade S *N* = 147	Aticama	10	S23
	Bahia Armenta	18	S6
	Los Barriles	6	S14
	Boquita	7	S20
	Cabo Pulmo	9	S26
	El Cajete	8	S2
	I. Cerralvo	8	S13
	Espiritu Santo (Cathedral)	8	S12
	Cabo San Lucas	6	S16
	Kino	8	S8
	Mulege	10	S5
	La Paz (Malecon)	10	S11
	Punta Mita	8	S21
	San Cosme	9	S4
	San Lucas	5	S7
	San Nicolas	9	S27
	Topolobampo	7	S25

### Geometric morphometric methods

We captured digital images of the dorsal side of each *Ligia* specimen using QCapture v. 3.1.2 with an Olympus QColor3 digital camera attached to an Olympus SZ61 stereomicroscope. All pereopods (i.e., legs) were removed prior to image capture to ensure the cephalon and pereon laid flat. Dissected pereopods were not used in the morphometric study. We defined the sex of each individual as gravid female (denoted as F), mature male (denoted as M), or others, which could be immature males or nongravid females (denoted as J). Gravid females harbor a ventral marsupium (i.e., thoracic pouch) with tens of embryos or yellow eggs. Mature males can be recognized by visual identification of gonopodia in the endopod of the 2nd pleopod.

**Figure 3:**
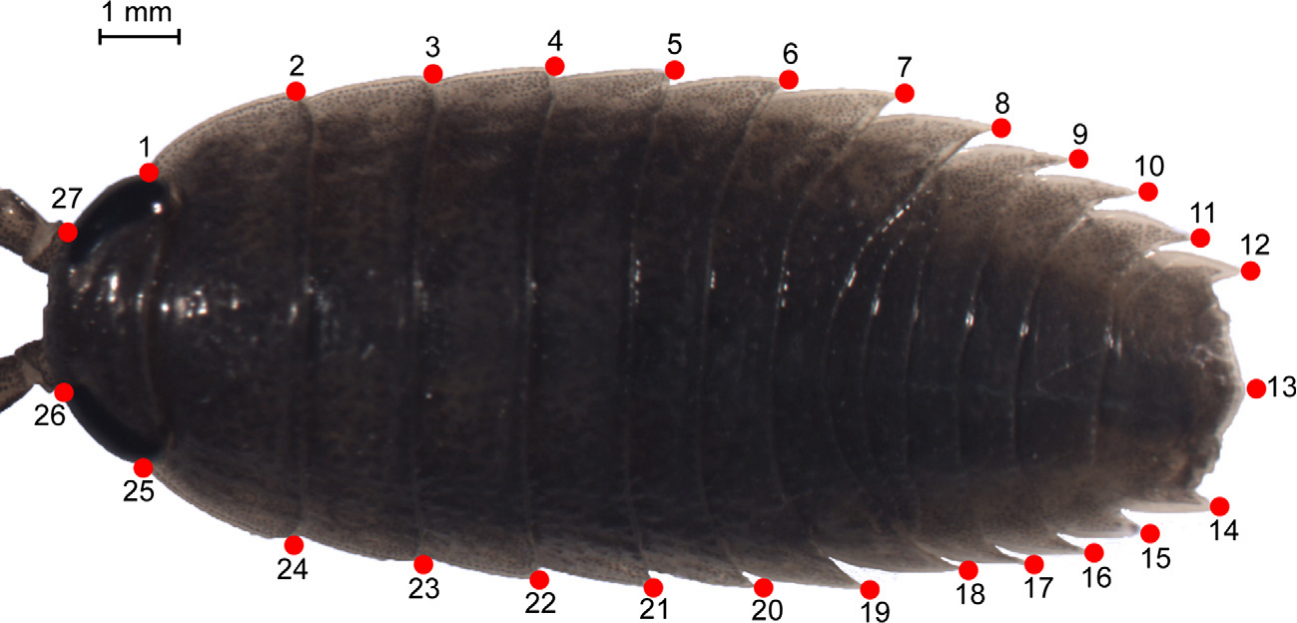
Placement in *Ligia* of landmarks (LMs) used in geometric morphometric analyses. LMs 1 and 25 represent the posterior margin of eye; LMs 2–11 and 15–24 are the lateral posterior tergite tips of each segment; LMs 12 and 14 are the lateral points of the pleotelson while LM 13 is the distal‐most point of the pleotelson; LMs 26 and 27 correspond to the inner most margin of the eyes.

We characterized body shape by digitizing 27 landmarks, using TpsDig v2.16 (Rohlf 2004), on the periphery of *Ligia* bodies (Fig. 3). Care was taken to include landmarks that captured taxonomically informative regions and that can be measured unambiguously. Landmarks were placed on medial and lateral boundaries of the eyes at the body periphery. These landmarks capture the relative size of the eyes and the distance between them, both characters used to distinguish *Ligia* species (Taiti et al. 2003). Landmarks were also placed on lateral posterior tergite tips to aid in characterizing relative width of each body segment and overall body shape, also important in *Ligia* taxonomy (Jackson 1922; Schultz and Johnson 1984; Lee 1994; Taiti et al. 2003; Khalaji‐Pirbalouty and Wägele 2010). Finally, landmarks were placed at the posterior tip and the lateral posterior points of the pleotelson. Relationships between these landmarks capture the shape of the pleotelson, another trait used in *Ligia* taxonomy (Schultz 1974; Taiti et al. 2003; Khalaji‐Pirbalouty and Wägele 2010).

As the body plan of *Ligia* is bilaterally symmetric, all but the pleotelson tip landmarks are anatomically homologous and should not be treated as independent in statistical analyses. We therefore reflected and averaged homologous landmarks across the midline (Zelditch et al. 2004), which was defined as a line connecting the pleotelson tip and the midpoint between the medial eye landmarks. Corrected landmarks were centered, scaled, and rotated, to best align with the consensus (i.e., average landmark configuration), using the method of generalized least squares, and projected to a flat (i.e., tangent) shape space using tpsRelw v1.49 (Rohlf 2006). We calculated principal components (PCs) of aligned coordinates to yield orthogonal shape variables. We also estimated log‐corrected centroid sizes (summed squared distances of landmarks from the centroid; Bookstein 1991) to use as a measure of body size.

### Statistical analyses

We conducted full factorial MANCOVA analyses of shape variables as a function of Lineage (i.e., *Clade*), Population nested within Lineage, Sex, Size, and all interactions, to discern the meaningful correlates of body shape. When interaction terms were not significant, we removed them from the model, hierarchically by order (i.e., from more complex to simpler), and repeated the analyses (Engqvist 2005). We estimated effect strengths for all terms in the final model by calculating partial eta‐squared values ($\eta_{P}^{2}$), which is the multivariate analog of R² in simple regression models (Tabachnick et al. 2001).

We explored differences among lineages, and among populations within lineages with discriminant function analyses (DFAs). To focus exclusively on between group differences, we first accounted for other predictors by conducting a preliminary MANCOVA based on Size and Sex and saving residual variation. Residuals were used in DFAs to focus discrimination solely on Lineage effects (Langerhans and DeWitt 2004). We tested whether all groups in our data shared a covariance matrix with the Box's M test. Although quadratic DFAs do not assume a homogeneous covariance matrix, singularities in the data matrix may prevent their correct use. In cases where neither linear nor quadratic DFAs could be correctly applied, we used regularized DFAs (Friedman 1989), as a compromise approach. We determined the best combination of *λ* (i.e., the degree of shrinkage of the individual class covariance matrix estimates toward the pooled estimate) and *γ* (i.e., the degree of shrinkage toward a multiple of the identity matrix) values by evaluating the risk of misclassification under several combinations of these parameters as suggested by Friedman (1989). We specified equal prior probabilities for each lineage in all DFAs analyses. Using this procedure, we attempted to assign individuals to (1) their clade of origin and (2) their population of origin within their corresponding clade. All results were validated using leave‐one‐out cross‐validation (LOOCV).

We tested for associations between phylogeny and morphological variation by estimating Pagel's *λ* (Pagel 1999) and Blomberg's *K* (Blomberg et al. 2003) for all shape variables, using Ives et al.'s (2007) method to account for multiple observations per terminal branch. The use of shape PCs in these analyses is justified, as they are aligned to the main axis of variation and maintain interobject distances (Perez et al. 2011). Both statistics provide a univariate measure of the strength of phylogenetic signal in the data, with values close to zero indicating no phylogenetic signal, and values close to one indicating the character has evolved under a Brownian motion (BM, i.e., phylogenetic signal explains the observed patterns). We tested whether observed *λ* values were statistically different from those expected under a null model (i.e., BM = 0) and a fully Brownian model (i.e., BM = 1) using a likelihood ratio test. In addition, we tested whether observed *K* values departed from the null hypothesis of no phylogenetic signal using 10,000 permutations (Blomberg et al. 2003). All computations were carried out using the Picante (Kembel et al. 2010) and GEIGER (Harmon et al. 2008) packages in R.

We also tested whether genetic divergence is related to multivariate morphological divergence. We estimated genetic distances from COI sequences published by Hurtado et al. (2010) using the K2P model in PAUP* (Swofford 2003). We calculated pairwise Euclidean distances for all localities on the residual variation (see above) using PopTools v. 3.2 (Hood 2010) in Microsoft^®^ Excel. We tested for correlations between COI K2P distances and Euclidean Morphological Distances (i.e., a Mantel test) and estimated *P*‐values by permutation. All statistical tests were carried out in JMP v9.0.1 (SAS Institute Inc., Cary, NC).

To visualize shape differences among lineages, we produced thin‐plate‐spline transformations (average shape deformation for each lineage from the consensus shape) of LM positions in tpsRegr v1.37 (Rohlf 2005) by entering our MANCOVA design matrix as the independent variable and our symmetrical landmark constellations as the dependent variables. We also produced transformations for the smallest and largest individuals for each clade and in the dataset overall to visualize shape differences between specimens of differing body size. We used these transformations to describe the general shapes of individuals within lineages and make comparisons among lineages.

## Results

### MANCOVA

Principal components analysis generated 24 nonzero eigenvectors. The first eleven PCs accounted for 95.4% of the variance and were included in subsequent analyses, whereas the other thirteen were discarded. The full factorial MANCOVA yielded no significant three‐ or four‐way interaction terms, which were removed prior to repeating the analysis. This simpler MANCOVA model (Table 2) yielded significant results for the effects of Population nested within Lineage, Lineage, Size, and for two interaction terms: Population x Sex [Lineage], and Population x Size [Lineage] (Table 2). Of these, the only effects with a partial eta square ($\eta_{P}^{2}$) value above 0.2 were Lineage, Size, and the interaction term Population × Size [Lineage] (Table 2). The main effect of Sex was not significant (Table 2).

**Table 2:** Results of multivariate analyses of overall body shape in *Ligia* isopods. Significant effects with a $\eta_{P}^{2}$ value >0.2 are indicated in bold

	*F* a	df_num_ b	df_den_ c	*P* d	$\eta_{P}^{2}$ e
Population [Lineage]	1.9977	242	3347.6	<0.0001	0.126
**Lineage**	**8.1999**	**77**	**2020.9**	**<0.0001**	**0.238**
Sex	1.2442	11	336	0.2563	0.039
**Size**	**41.776**	**11**	**336**	**<0.0001**	**0.578**
Population*Sex [Lineage]	1.4006	495	3631.4	<0.0001	0.160
Population*Size [Lineage]	**2.0240**	**495**	**3631.4**	**<0.0001**	**0.216**

a Approximate *F*‐statistic.

b Degrees of freedom for numerator.

c Degrees of freedom for denominator.

d *P*‐value for the corresponding test.

e Effect size as measured by eta squared (partial variance explained by variable).

### Discriminant function analyses

**Figure 4:**
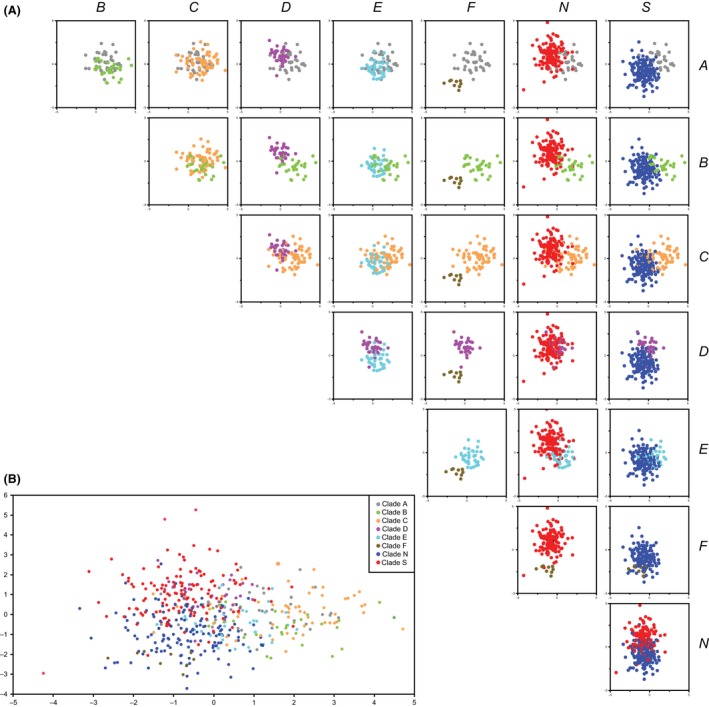
Results of discriminant function analyses (DFA) on morphology for *Ligia* lineages. Lineages are identified by color, which correspond to those in other figures and in Hurtado et al. (2010). Canonical plots are shown for each pairwise comparison between *Ligia* lineages (Panel A) and for the overall dataset (Panel B). The first and second canonical axes explained 43.13% and 32.20% of the variance, respectively.

Results of the Box's M test indicated that covariance matrices are heterogeneous across lineages (Box's M: 1123.2, df_error_ = 72611.9, *P *<* *0.0001), suggesting linear DFAs were inappropriate for our dataset. We could not implement quadratic DFAs, however, due to singularities in our data matrix. Therefore, we implemented regularized DFAs. We used *λ* and *γ* values of 0.1 in the final analyses, as low values for these parameters are recommended when covariances are different, data are abundant, and when variables may be correlated (SAS Institute Inc. 2010). Also, these values produced the lowest misclassification rates in preliminary analyses under a variety of *λ* and *γ* combinations, another criterion for parameter selection (Friedman 1989). Regularized DFAs of residuals indicated significant differences between Lineages (Λ = 0.165, df_error_ = 2847.8, *P* < 0.0001). No distinct clusters, however, were seen in canonical plots, which may be explained by the extensive overlap between most lineages in pairwise comparisons (Fig. 4).

Initially, a correct assignment of individuals to their lineage of origin was achieved in 72.8% of cases, but a considerably smaller classification success rate was obtained using leave‐one‐out cross‐validation (LOOCV): 57.9%. Per‐lineage validated correct classification rates were as follows: 57.9% for *Clade A*; 38.7% for *Clade B*; 44.3% for *Clade C*; 69.7% for *Clade D*; 59.5% for *Clade E*; 44.4% for *Clade F*; 59.1% for *Clade N*; and 64.4% for *Clade S*. Most misclassified individuals were assigned to geographically nearby lineages. A full breakdown of classification results is shown in Table 3. We observed similar patterns under different combinations of *λ* and *γ* between 0.1 and 0.5 (data not shown).

**Table 3:** Classification rates for discriminant function analyses of *Ligia* isopods to lineage of origin. Rows indicate actual clade of origin, while columns indicate predicted membership. We present the percentage of individuals correctly assigned to their clade of origin (boldface) for the original model (upper) and LOOCV rates (lower)

	*A*	*B*	*C*	*D*	*E*	*F*	*N*	*S*
*A*	**73.68**	2.63	18.42	0.00	2.63	0.00	0.00	2.63
	**57.89**	5.26	26.32	0.00	5.26	0.00	0.00	5.26
*B*	3.23	**83.87**	3.23	6.45	3.23	0.00	0.00	0.00
	16.13	**38.71**	29.03	6.45	3.23	0.00	3.23	3.23
*C*	14.75	4.92	**63.93**	1.64	8.20	0.00	6.56	0.00
	19.67	11.48	**44.26**	1.64	11.48	0.00	8.20	3.28
*D*	0.00	0.00	0.00	**100.00**	0.00	0.00	0.00	0.00
	0.00	0.00	9.09	**69.70**	6.06	0.00	9.09	6.06
*E*	0.00	2.70	5.41	2.70	**86.49**	0.00	0.00	2.70
	0.00	5.41	13.51	2.70	**59.46**	0.00	8.11	10.81
*F*	0.00	0.00	0.00	0.00	0.00	**100.00**	0.00	0.00
	0.00	0.00	0.00	0.00	11.11	**44.44**	0.00	44.44
*N*	2.92	2.19	0.00	4.38	9.49	0.00	**64.96**	16.06
	2.92	2.19	2.92	4.38	9.49	0.00	**59.12**	18.98
*S*	2.05	5.48	1.37	4.79	8.90	2.05	5.48	**69.86**
	2.74	6.16	1.37	5.48	9.59	2.05	8.22	**64.38**

We conducted regularized DFAs (*λ *= 0.1, *γ *= 0.1) assigning individuals to the localities of origin for each lineage separately. All lineages and localities were used except *Clade F*, as it only consisted of one locality (Puerto Vallarta). All DFAs were significant (Table 4), but the percentage of individuals correctly assigned to their locality within each clade after LOOCV was low (range: 24.3–61.7%; Table 4), compared to before LOOCV (range: 97–100%).

**Table 4:** Results of regularized DFAs (*λ *= 0.1, *γ *= 0.1) assigning individuals to localities of origin for each lineage separately

	Λ^a^	df_error_ b	*P* c	Initial^d^	LOOCV^e^	*k* f
Clade A	0.13	71.4	<0.003	100.0	44.7	4
Clade B	0.20	36.0	<0.031	100.0	61.3	3
Clade C	0.03	240.9	<0.0001	98.4	42.6	7
Clade D	0.21	40.0	<0.017	97.0	54.6	3
Clade E	0.07	33.0	<0.0001	100.0	24.3	4
Clade N	0.02	975.5	<0.0001	97.1	25.6	14
Clade S	0.01	1128.3	<0.0001	97.3	37.0	17

a Wilk's Lambda value.

b Degrees of freedom.

c *P*‐value.

d Initial rate of correct classification to locality within clade.

e Cross‐validated rate of correct classification to locality within clade.

f Number of localities.

### Tests of phylogenetic signal

We did not detect widespread evidence of phylogenetic structure in the shape variables using either *λ* or *K* statistics, but did notice a specific instance of structure related to the fifth shape PC (Table 5). This last result, however, appeared to be spurious, as no obvious differences were observed between lineages upon inspection. Although the statistical power of these tests is maximized when *N *>* *20 (Pagel 1999; Blomberg et al. 2003), Pagel's *λ* values are robust to the number of taxa included, whereas Blomberg's *K* values decrease as additional taxa are included (Münkemüller et al. 2012). Exploratory analyses incorporating guide trees with major clades subdivided into component lineages produced results consistent with these expectations and concordant with the results presented herein. We do not present these results, as the statistical significance of *K* values cannot be inferred using unresolved guide trees (Kembel et al. 2010).

**Table 5:** Results of analyses of phylogenetic signal for shape variables (i.e., relative warps) included in multivariate analyses of shape

	Blomberg's *K*	P^a^	Pagel's *λ*	M.L. (lnl)^b^	*P* = 0^c^	*P* = 1^c^
RW1	0.335	0.116	1.0E‐07	21.7	1.000	0.069
RW2	0.067	0.491	2.0E‐05	24.8	1.000	0.000
RW3	0.316	0.093	1.0E‐07	24.8	1.000	0.064
RW4	0.016	0.948	4.5E‐05	25.2	1.000	0.000
RW5	0.825	0.062	1.0E+00	29.6	0.080	1.000
RW6	0.034	0.663	1.1E‐05	33.1	1.000	0.000
RW7	0.022	0.799	1.7E‐06	34.4	1.000	0.000
RW8	0.012	0.975	8.0E‐02	35.5	0.862	0.000
RW9	0.009	0.982	7.0E‐06	35.2	1.000	0.000
RW10	0.010	0.938	7.9E‐06	32.8	1.000	0.000
RW11	0.007	0.985	4.8E‐06	36.0	1.000	0.000

a *P*‐value for observed Blomberg's *K* value based on 10,000 randomizations.

b Likelihood of observed Pagel's *λ* value.

c probability observed *λ* diverges from a null (BM = 0) and fully Brownian model (BM = 1).

**Figure 5:**
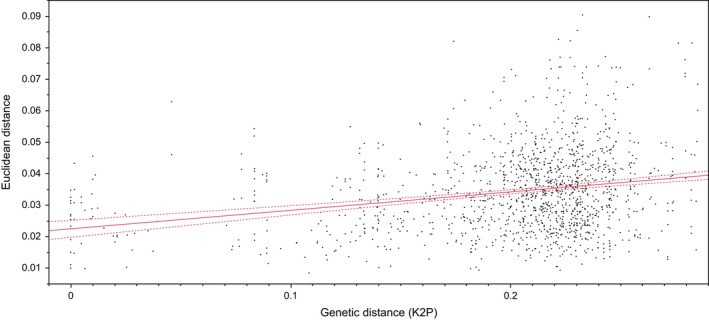
Correlation between pairwise genetic distances and Euclidean distances. The solid line indicates best‐fit line, and dotted line indicates confidence limits at 95%.

K2P genetic distances ranged from 0.00 to 28.5% (mean = 20.2%, median = 21.6%). Euclidean distances in the morphological dataset ranged from 0.008 to 0.09 (*μ *= 0.034, Median = 0.033). Lower dispersal of Euclidean distance values was observed at smaller genetic divergences. Regression of pairwise morphological distances against pairwise K2P genetic distances (Fig. 5) was significant (*F *=* *79.0491, df_error_ = 1375, *P *<* *0.0001); however, the *R* ^2^ value suggested a poor fit between the data and the model (*R* ^2 ^= 0.054). Mantel tests are known to be afflicted by high type‐I error rates (Lapointe and Legendre 1995; Oberrath and Bohning‐Gaese 2001; Nunn et al. 2006), and their use in phylogenetic comparative analyses has been discouraged (Harmon and Glor 2010). Nonetheless, we incorporated Mantel tests to determine whether the combination of all shape variables produced patterns different than those seen by evaluating shape variables independently.

### Visualization of shape variation in *Ligia*

**Figure 6:**
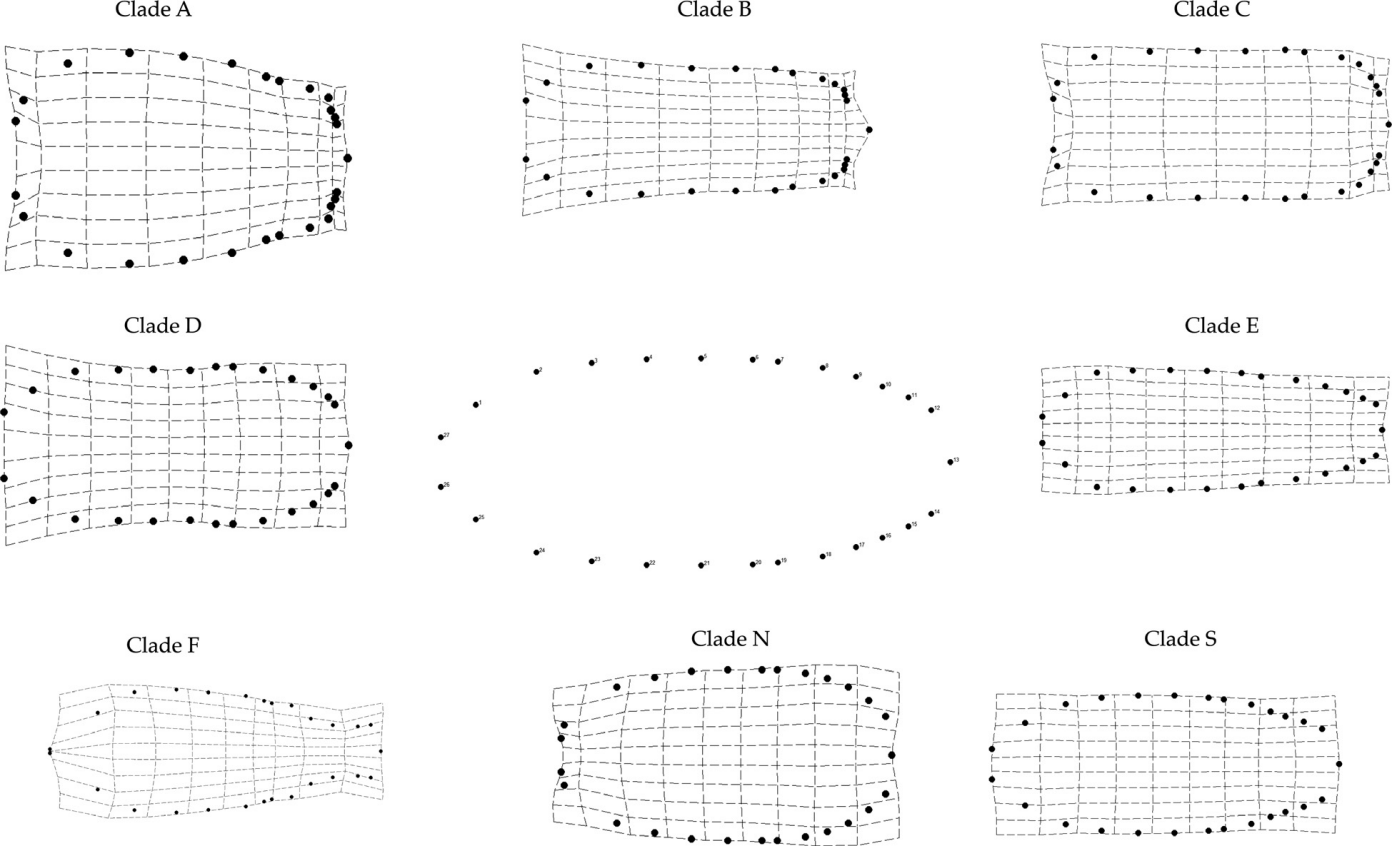
Thin‐plate‐spline transformations of LM positions for each *Ligia* lineage. Transformations are shown at 10*X natural range to aid visualization.

We present thin‐plate‐spline transformations for all major clades using vector magnifications of 10× for ease of comparison (Fig. 6). Because such magnifications have as much to do with statistical power as with the magnitude of effects, we selected individuals from each clade with the highest canonical score and provide those images as supplemental material (Fig. S1). Between‐clade differences appear most pronounced in the cephalon, pleotelson, and midbody regions. *Ligia* individuals in *Clade A* exhibit an enlarged cephalon with small eyes. Their pleotelson is compressed, with the lateral posterior and the distal‐most point almost parallel. *Clade C* has a pleotelson similar to that of *Clade A*; however, a somewhat rectangular body shape and small eyes with no enlarged cephalon may distinguish individuals from *Clade C*. Individuals in *Clade B* are characterized by an oval body shape with a normally sized cephalon and medium‐sized eyes. As in *Clade A*, the pleotelson is compressed; however, the distal‐most point protrudes well beyond the lateral posterior points. *Clade D* exhibits a slight invagination in the midbody region and medium‐sized eyes on a regular cephalon. Their pleotelson appears less compressed than other clades, with the exception of *Clade E*. Although exhibiting a similar pleotelson, *Clade E* has no midbody invagination. Also, individuals from this clade exhibit medium‐sized eyes with a slightly larger cephalon than the rest of the body (i.e., the body tapers posteriorly). *Clade S* exhibits a body shape similar to those in *Clade E*; however, the body does not appear to taper, and the distal point of the pleotelson appears to protrude more extensively than in *Clade E*. *Clade F* specimens have very large eyes, with a drastic invagination in the segments prior to the pleotelson. *Clade N* has small eyes, with an oval body shape, a noncompressed pleotelson, and a large 1st segment.

**Figure 7:**
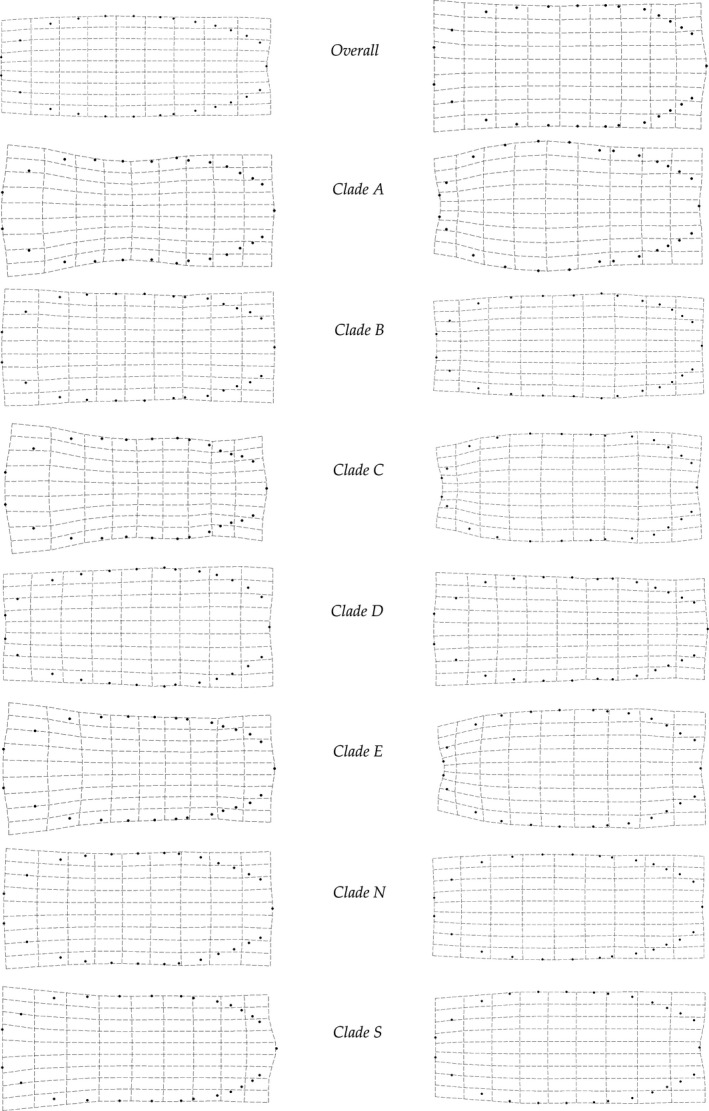
Thin‐plate‐spline transformations of LM positions for *Ligia* size minima (left) and maxima (right) for the overall dataset, and for each lineage. *Clade F* is not presented due to small sample sizes. Transformations are shown at 3*X the natural range to aid visualization.

We also present thin‐plate‐spline transformations for the largest and smallest individuals for both the overall dataset (i.e., Size effect) and for each lineage (i.e., Lineage*Size) (Fig. 7). In general, larger individuals exhibit a broader body and smaller eyes (relative to the total body size), with a distal point of the pleotelson that is slightly more protruding. All lineages appear to exhibit similar patterns, differing mostly in the magnitude of the effect. Individuals in clades *A*,* C*,* D*, and *E* exhibit the most obvious allometric effects (Fig. 7). Much subtler differences are observed between the large and small individuals in clades *B*,* N*, and *S* (Fig. 7).

## Discussion

Taken together, our results suggest that *L. occidentalis s. l*. experiences morphological stasis. We detected significant differences in body shape among the major clades (variable Lineage) of *L. occidentalis s. l*., and among populations within these clades, but only the effect size of Lineage appears to be important (i.e., $\eta_{P}^{2}$ > 0.2). Nonetheless, neither lineages nor populations can be discriminated on the basis of body shape, as correct classification rates of cross‐validated DFAs were low (overall among lineages = 57.9%; range among populations within lineages = 24.3–61.3%). Such low rates of correct classification may be attributable to a large overlap of body shape among clades, as revealed by the canonical plots (Fig. 4). The much higher rates of correct classification prior to cross‐validation implies that they were inflated, possibly reflecting over‐fitting (Kovarovic et al. 2011). Failure to perform DFA cross‐validation can produce severely biased results (Kovarovic et al. 2011) and thereby lead the investigator to erroneously conclude that the lineages under question can be distinguished with high accuracy. Unfortunately, studies that employ DFA often fail to report cross‐validated results (e.g., Adams et al. 2014). Our results underscore the importance of cross‐validation when conducting DFAs.

Extensive overlap among clades suggests body shape variation in *L. occidentalis s. l*. is highly constrained. It is possible that body shape variation can only occur within a narrow morphospace, henceforth the overlap among clades; and/or that morphological differentiation does occur, albeit at a very slow rate compared to genetic differentiation. Although the small slope of the Euclidean morphological distance versus genetic distance regression is suggestive of this pattern (Fig. 5), the poor fit of the model prevents robust inferences. Nonetheless, the largest Euclidean distances were observed at larger genetic distances. Due to the deep genetic divergences among the main lineages of *L. occidentalis s. l*., with some splits probably dating to the Miocene (Hurtado et al. 2010), the absence of strong body shape divergence is unlikely explained by a lack of genetic variation stemming from failure of mutations to arise.

A similar finding of significantly different, but not fully diagnostic body shapes, was observed among three genetically divergent lineages of *L. hawaiensis*, an endemic of the Hawaiian archipelago (Santamaria et al. 2013). Although divergences among the three *L. hawaiensis* lineages are smaller than the deepest divergences among *L. occidentalis s. l*. lineages, they probably represent several million years of separation. Other cryptic species complexes of *Ligia* that include highly divergent lineages, which also appear to represent millions of years of separation, have been discovered in the Caribbean (Santamaria et al. 2014) and the Mediterranean (Hurtado et al. unpublished results). Despite a greater phylogenetic depth, considerable overlap in body shapes occurs between *L. occidentalis s. l*. versus *L. hawaiensis* (Fig. S2). Morphological stasis, thus, appears to be a common phenomenon within *Ligia*; a group that is at least ∼110 Ma‐old based on the fossil record (Broly et al. 2013). Most *Ligia* species are coastal (Taiti et al. 2003; Broly et al. 2013) and exhibit morphological, physiological, and behavioral characteristics that are intermediate between ancestral marine and fully terrestrial isopods (Carefoot and Taylor 1995). Ligiidae occupy the most basal position within Oniscidea (Erhard 1998; Schmidt 2008), and it is believed that the ancestor of oniscideans had ligiid‐like characters (Carefoot and Taylor 1995; Schmidt 2008). The traits that have allowed *Ligia* isopods to persist and diversify in their supralittoral environment may have contributed to constrain body shape evolution.

Strong stabilizing selection on body shape could be exerted by one or more inherent features of the intertidal habitat occupied by *L. occidentalis s. l*. (and other coastal *Ligia*). This selection must be efficient enough to counter the effect of drift, which is expected to be strong in many populations, due to their apparently small size and genetic isolation (Hurtado et al. 2010; Eberl et al. 2013). These isopods occupy a very narrow vertical range of rocky shores (i.e., from the water line to the supralittoral), where they track the shifting water line, and actively avoid the open sea. Because of their extremely low desiccation resistance, they must remain close to water, where they can take up water from droplets and puddles by capillarity and from water vapor directly from the air (Carefoot and Taylor 1995). They tend to be more active at night and remain hidden under rocks and in crevices during much of the day (Carefoot and Taylor 1995; Hurtado et al. 2010), presumably to minimize water loss and avoid terrestrial/aerial predators (e.g., lizards, birds, and invertebrates; Hurtado et al. 2010; Grismer 1994; Brusca 1980). Their coloration enables them to blend in with the rocky substrate. Locomotion of these isopods is well adapted to the rocky substrate, where they dash for cover when threatened. On sand, however, their locomotion is extremely limited and their coloration is more conspicuous, rendering them highly vulnerable to predators. In addition, they may be more susceptible to wave action and desiccation, due to the lack of cover, in sandy substrate. Despite variation in the nature of the rocky beach type (i.e., gravel, cobbles, pebbles, boulders, and rocky bench; all of which are occupied by *L. occidentalis s. l*.), the rocky substrate may represent a rather homogeneous habitat that promotes the retention of a highly conserved body morphology. Accordingly, *L. occidentalis s. l*. appears to exhibit niche conservatism, at least in terms of substrate type (rocky) as it relates to body shape. Due to its effect on locomotory function, substrate type can be a critical determinant of morphology (e.g., Losos et al. 1997; Vervust et al. 2007; Goodman et al. 2008). *Tylos*, another cosmopolitan supralittoral endemic isopod genus, but that is restricted to sandy substrates, also exhibits high levels of cryptic diversity (Hurtado et al. 2013, 2014), and presumably body shape stasis. Therefore, this phenomenon may be common among permanent members of the supralittoral environment.

Body shape variation in terrestrial isopods (i.e., Oniscidea) is suggested to be constrained by a limited number of constructional pathways (Schmalfuss 1984). Accordingly, most of the ~3700 species terrestrial isopods (Sfenthourakis and Taiti 2015) can be classified into five major functional categories of skeletal construction, which are correlated to ecological strategies and behavioral patterns: runners; clingers; rollers (e.g., *Tylos*); spiny forms; and creepers (Schmalfuss 1984). Runners dash when threatened to quickly hide under rocks or in crevices and are characterized by a narrow body, smooth tergites, long and strong pereopods, and a convex hind‐margin of first epimeres. Tropical and subtropical species of *Ligia*, such as *L. occidentalis s. l*., are typically runners, whereas some temperate species of *Ligia* are suggested to be clingers (Schmalfuss 1984). Clingers, when threatened, tend to remain motionless clinging tightly to a solid substrate. Clingers differ from runners by a broader body, shorter pereopods, and a concave hind‐margin of first epimeres (Schmalfuss 1984). Some body shape differences, thus, appear to occur between these two ecomorphs in *Ligia*.

Similarities in body shape among divergent lineages of *L. occidentalis s. l*. may be influenced by phylogenetic relatedness, geographic proximity (which may imply exposure to more similar environmental/ecological conditions), or stochasticity. We note, however, that phylogenetic relatedness and geographic proximity are highly confounded in *L. occidentalis s. l*., with phylogenetically closer lineages generally having adjacent distributions (Eberl et al. 2013; Hurtado et al. 2010; Hurtado et al., unpublished results). *Clade A* is sister to *Clade BCDE*; relationships within this last group are (*B* (*E* (*C D*))). Similarly, *Clade N* is sister to *Clade S*, and both occupy adjacent distributions in the Gulf of California, the former in the northern Gulf, whereas the latter in the southern Gulf. *Clade F* is a highly divergent lineage, whose distribution overlaps with that of *Clade S*. The relationships among the highly divergent *ABCDE*,* NS*, and *F* clades are uncertain.

Even though phylogenetic relatedness and geographic proximity are confounded, data on the clades to which misclassified individuals were assigned (Table 3) suggest that geographic proximity influences the degree of body shape similarity among clades. Individuals from geographically adjacent clades appear to have more similar body shapes. For example, the majority of the misclassified individuals by cross‐validated DFA from *Clade A* were placed in *Clade C*, and vice versa. Most of the localities sampled from clades *A* and *C* are in the Northern Channel Islands. Therefore, geographic proximity appears to have a stronger influence than phylogenetic relatedness on the misclassification of individuals from clades *A* and *C* (*A* is sister to *BCDE*). Similar situations are observed for misclassified individuals of the other clades with the exception of *Clade E*. A noteworthy case is that of the misclassified individuals of *Clade F*, which were classified as members of *Clade S* in equal proportion to the correctly assigned individuals (i.e., 44.4%; Table 3). Clades *F* and *S* are phylogenetically distant, but the localities of *Clade F* examined are geographically nested within those of *Clade S*. The pairwise canonical plots show a remarkable separation between *Clade F* and all other clades, except *Clade S*, with which it exhibits complete overlap on the two major dimensions of among group variance (Fig. 4). Another example is that of *Clade S*, for which most misclassified individuals were assigned to geographically nearby clades *N* and *E*. Whereas clades *N* and *S* are sister lineages, *Clade E* is distantly related. Misclassification of individuals in *Clade E*, however, appears to be more stochastic, with few individuals being incorrectly assigned to the geographically adjacent *Clade D*. The lack of strong signal from phylogeny and genetic distance on body shape variation is consistent with the apparent effect of geographical proximity described above.

The apparent influence of geographic proximity on body shape similarity suggests, however, that the environment might impose at least some weak directional selection on shape variation. For example, the similarity between clades *A* and *C*, most of which were sampled on the Northern California Channel Islands, despite the marked differences in SST among insular localities occupied by the two clades (Eberl et al. 2013), suggests that insular ecological factors may be relevant to body shape (e.g., different or fewer terrestrial predators may be present in the islands). Determining the influence of extrinsic and intrinsic factors on body shape variation in *L. occidentalis s. l*. will require studies of ecological parameters, as well as of the genetic architecture of body shape, including an assessment of phenotypic plasticity (Wake 1991; Schlichting and Pigliucci 1998). This would allow evaluation of mechanisms other than stabilizing selection on body shape per se that may limit the evolution of this trait in *L. occidentalis s. l*. (e.g., genetic and developmental constraints, and stabilizing selection on traits correlated to body shape; Futuyma 2010).

We detected allometric effects on the overall body shape of *L. occidentalis s. l*., with $\eta_{P}^{2}$ values (Table 2) suggesting body size to be the strongest determinant of overall body shape. In general, larger *Ligia* individuals (usually males) exhibit a disproportionately wider body than smaller individuals, a pattern reported for *L. pallasii* (Carefoot 1973). In *L. hawaiensis*, however, larger individuals exhibit a more elongated body with relatively smaller head than smaller individuals (Santamaria et al. 2013). Thin‐plate‐spline visualizations suggest widening of the body is not uniform across *L. occidentalis s. l*. lineages. Developmental (Stern and Emlen 1999) or ecological differences (Pfennig 1992) may be responsible for the differences in the magnitude of this effect observed in *L. occidentalis s. l*. Three of the lineages (*C*,* D*,* E*) exhibiting the deepest widening of the body constitute a well‐supported monophyletic clade, with those exhibiting no obvious allometric effects (*N* and *S*) also forming a monophyletic group (Hurtado et al. 2010). These patterns may be indicative of the shared evolutionary history of these lineages and may be due to shared developmental constrains. On the other hand, environmental factors may be at play. Growth rates of isopods are known to be affected by environmental factors such as temperature (Strong and Daborn 1980; Holdich and Tolba 1981; Donker et al. 1998), food availability (Reichle 1968), and exposure to pollutants (Donker et al. 1993). In general, lineages in the colder Pacific Ocean (*A*,* C*,* D*,* E*) exhibited obvious allometric effects, whereas those in the warmer Gulf of California (*N*,* S*) did not. Furthermore, we observed some obvious allometric effects in some *Clade N* localities (N7, N9, N12), suggesting that differences in allometric effects on body shape may also correspond to ecological factors and not phylogenetic trajectory. As the distribution of *L. occidentalis s. l*. lineages closely matches changes in sea surface temperatures (Eberl et al. 2013), additional work is needed to establish the contributions of ecological differences and phylogenetic relatedness on the observed differences in the magnitude of allometric changes.

*Ligia occidentalis s. l*. appears to truly represent a hypercryptic species complex, lacking diagnostic morphological differences among putative species. Preliminary examination for differences among lineages based on traditional characters used in *Ligia* has failed to reveal obvious differences. In addition, one of us (CAS) has preliminarily examined SEM micrographs of the appendix masculina and 7th pereopod of individuals representing the main clades, and has not found clear differences. The apparent hypercryptic nature of this taxon brings about challenges for the conservation of a unique and highly vulnerable biodiversity. The current recognition of a single widely distributed species hampers preservation efforts. Unique cryptic lineages have very restricted geographic ranges, usually constrained to discrete beaches, with some that appear to have small population sizes. The rocky supralittoral habitat in the range of *L. occidentalis s. l*. is very vulnerable to destruction and modification by human activities (e.g., construction and pollution), which have increased as human populations and tourism expand (Hurtado et al. 2010, 2013); and populations of this isopod across its range have been shown to accumulate toxic contaminants from anthropogenic activities (García‐Hernández et al. 2015). Storms and hurricanes may sweep local populations (Hurtado, personal observation), whereas, in the long term, sea level rise could also threaten the persistence of populations. In the absence of morphological differences that can be taxonomically diagnostic, we suggest the use of molecular information to taxonomically classify *L. occidentalis s. l*. in a way that reflects and helps protect its unique diversity. Defining species based on molecular data alone has been conducted in other cryptic species complexes (Dumas et al. 2015; Wade et al. 2015). In *L. occidentalis s. l.,* the process may be facilitated by the discrete geographic distribution of lineages. We suggest the use of a divergence cutoff to assign species and subspecies, which should aid in the conservation of the rich biodiversity found within this clade.

## Conflict of Interest

None declared.

## Data accessibility

Morphometrics datasets and pictures are available in Dryad Digital Repository (http://dx.doi.org/10.5061/dryad.cj32g).

## Supporting information


**Figure S1.** Photographs of individuals with the highest canonical score in Discriminant Function Analyses (DFA) for each *L. occidentalis* lineage.
**Figure S2.** Results of Discriminant Function Analyses (DFA) for *Ligia occidentalis* and *Ligia hawaiensis*. Species are identified by colour and shape, with red circles corresponding to *L. occidentalis* samples and blue triangles to *L. hawaiensis*.
