# Supplementary material for: Constrained body shape among highly genetically divergent allopatric lineages of the supralittoral isopod Ligia occidentalis (Oniscidea)
Source: Ecol Evol. 2016 Feb 9;6(5):1537–54. doi: 10.1002/ece3.1984 (PMC4747314; doi:10.1002/ece3.1984)
Supplement: Supplementary file 1 — Figure S1. Photographs of individuals with the highest canonical score in Discriminant Function Analyses (DFA) for each L. occidentalis lineage. Figure S2. Results of Discriminant Function Analyses (DFA) for Ligia occidentalis and Ligia hawaiensis. Species are identified by colour and shape, with red circles corresponding to L. occidentalis samples and blue triangles to L. hawaiensis. [file ECE3-6-1537-s001.docx]

**Supporting Information**

Figure S1. Photographs of individuals with the highest canonical score in Discriminant Function Analyses (DFA) for each *L. occidentalis* lineage.

Figure S2. Results of Discriminant Function Analyses (DFA) for *Ligia occidentalis* and *Ligia hawaiensis*. Species are identified by colour and shape, with red circles corresponding to *L. occidentalis* samples and blue triangles to *L. hawaiensis*.
